# Phase 2 study of the efficacy and safety of ponsegromab in patients with cancer cachexia: PROACC‐1 study design

**DOI:** 10.1002/jcsm.13435

**Published:** 2024-03-18

**Authors:** John D. Groarke, Jeffrey Crawford, Susie M. Collins, Shannon L. Lubaczewski, Danna M. Breen, Magdalena A. Harrington, Ira Jacobs, Ruolun Qiu, James Revkin, Michelle I. Rossulek, Aditi R. Saxena

**Affiliations:** ^1^ Internal Medicine Research Unit Pfizer Inc Cambridge MA USA; ^2^ Duke Cancer Institute Duke University Medical Center Durham NC USA; ^3^ Global Biometrics and Data Management Pfizer R&D UK Ltd Sandwich Kent UK; ^4^ Early Clinical Development and Biomedicine Artificial Intelligence Pfizer Inc Collegeville PA USA; ^5^ Global Access and Value Pfizer Inc Cambridge MA USA; ^6^ Global Product Development Pfizer Inc New York NY USA; ^7^ Clinical Pharmacology Pfizer Inc Cambridge MA USA

**Keywords:** cachexia, cancer, GDF‐15, ponsegromab, weight loss

## Abstract

**Background:**

Cancer cachexia is a multifactorial metabolic wasting syndrome characterized by anorexia, unintentional loss of weight involving both skeletal muscle and adipose tissues, progressive functional impairment and reduced survival. Therapeutic strategies for this serious condition are very limited. Growth differentiation factor 15 (GDF‐15) is a cytokine that is implicated in cancer cachexia and may represent both a biomarker of cancer cachexia and a potential therapeutic target. Ponsegromab is a potent and selective humanized monoclonal antibody that inhibits GDF‐15‐mediated signalling. Preclinical and preliminary phase 1 data suggest that ponsegromab‐mediated inactivation of circulating GDF‐15 may lead to improvement in key characteristics of cachexia. The primary objective of this phase 2 study is to assess the effect of ponsegromab on body weight in patients with cancer, cachexia and elevated GDF‐15 concentrations. Secondary objectives include assessing physical activity, physical function, actigraphy, appetite, nausea and vomiting, fatigue and safety. Exploratory objectives include evaluating pharmacokinetics, pharmacodynamics, immunogenicity, lumbar skeletal muscle index and Response Evaluation Criteria in Solid Tumors.

**Methods:**

Approximately 168 adults with non‐small‐cell lung, pancreatic or colorectal cancers who have cachexia and elevated GDF‐15 concentrations will be randomized in a double‐blind, placebo‐controlled study (NCT05546476). Participants meeting eligibility criteria will be randomized 1:1:1:1 to one of three dose groups of ponsegromab (100, 200 or 400 mg) or matching placebo administered subcutaneously every 4 weeks for an initial 12‐week treatment period. This is followed by optional open‐label treatment with ponsegromab of 400 mg administered every 4 weeks for up to 1 year. The primary endpoint is mean change from baseline in body weight at Week 12. A mixed model for repeated measures followed by a Bayesian E_max_ model will be used for the primary analysis. Secondary endpoints include physical activity, physical function and actigraphy measured by remote digital sensors; patient‐reported appetite‐related symptoms assessed by Functional Assessment of Anorexia‐Cachexia Therapy subscale scores; anorexia/appetite, nausea and vomiting, and fatigue evaluated according to questions from the Cancer‐Related Cachexia Symptom Diary; and incidence of adverse events, safety laboratory tests, vital signs and electrocardiogram abnormalities.

**Perspective:**

Cancer‐related cachexia is an area of significant unmet medical need. This study will support the clinical development of ponsegromab as a novel inhibitor of GDF‐15, which may ameliorate key pathologies of cancer cachexia to improve patient symptoms, functionality and quality of life.

**Trial registration:**

ClinicalTrials.gov ID: NCT05546476.

## Introduction

Cachexia is a multifactorial metabolic wasting syndrome, characterized by anorexia and unintentional ongoing loss of skeletal muscle mass (with or without loss of adipose tissue), that cannot be reversed by nutritional support and leads to progressive functional impairment.^1^, ^2^, ^3^, ^4^ Cachexia is common in patients with cancer, with an estimated prevalence in the United States of approximately 30% across all cancer types and approximately 32%, 37% and 46% in patients with colorectal cancer (CRC), lung cancer and pancreatic cancer (PANC), respectively.^5^ Furthermore, approximately half of patients with advanced cancer are estimated to develop cachexia.^1^ The gradual worsening of cachexia as cancer progresses can be highly burdensome, leading to physical disability, fatigue, diminished quality of life, increased susceptibility to toxicities of anticancer therapies and reduced survival.^1^, ^2^, ^3^ Recently published guidelines emphasized that there is no recommended pharmacologic standard of care for patients with cancer‐associated cachexia.^3^ Herein, we present the rationale and design for the **P**atient **R**eported **O**utcomes and **A**ctivity in **C**an**c**er (PROACC‐1) study (NCT05546476), which is a multicentre, randomized, double‐blind, placebo‐controlled study that will assess the effect of ponsegromab, a humanized monoclonal antibody that inhibits growth differentiation factor 15 (GDF‐15), in patients with cancer, cachexia and elevated circulating levels of GDF‐15.

## Rationale

### Role of growth differentiation factor 15 in cancer cachexia

GDF‐15 is a cytokine of the glial cell line‐derived neurotrophic factor family within the TGF‐β (transforming growth factor beta) superfamily.^6^ GDF‐15 functions through the glial cell‐derived neurotrophic factor family receptor α‐like (GFRAL) receptor localized in the hindbrain.^6^ GDF‐15 acts as a global regulator of stress in response to stimuli of varying origin, including environmental, medical therapies (including chemotherapy and radiation therapy) and chronic diseases (e.g., cancer and heart failure).^6^, ^7^, ^8^, ^9^ Preclinical studies in mouse models and non‐human primates implicate GDF‐15 in cancer cachexia. GDF‐15 decreases food intake, increases energy expenditure and decreases body weight in rodents and non‐human primates through GFRAL receptors.^10^, ^11^, ^12^, ^13^, ^14^, ^15^ Consistent with the negative energy balance phenotype, GDF‐15 also induces functional impairment in rodents as shown by reductions in voluntary wheel running activity.^14^, ^15^

Elevated serum levels of GDF‐15 are reported in patients with various types of cancer and are associated with weight loss and poor survival.^9^, ^16^, ^17^ In addition, GDF‐15 levels are elevated in patients on platinum‐based chemotherapy.^8^ A significant association between circulating GDF‐15 and loss of body weight, skeletal muscle and adipose tissue was identified at relapse in an exploratory proteomic analysis of circulating putative mediators of cachexia, performed in a subset of 110 individuals from TRACERx, a prospective multicentre study of participants with primary non‐small‐cell lung cancer (NSCLC).^4^ Collectively, these data support the potential therapeutic relevance of targeting GDF‐15 in the management of cancer cachexia.

### Ponsegromab as a novel anti‐growth differentiation factor 15 therapy in development

Ponsegromab (PF‐06946860) is a potent and highly selective humanized monoclonal antibody directed against GDF‐15 that is currently being investigated in patients with cancer, cachexia and elevated circulating concentrations of GDF‐15. Ponsegromab is delivered subcutaneously (SC) and binds to circulating GDF‐15 protein, thereby preventing its interaction with GFRAL. It is hypothesized that cachexia in many types of cancer, including NSCLC, CRC and PANC, is largely mediated via GDF‐15 and that suppression of GDF‐15 may improve cachexia‐related symptoms, including anorexia, leading to unintended weight loss, fatigue and impaired mobility. By inhibiting GDF‐15‐mediated signalling, ponsegromab has the potential to ameliorate the key pathologies of cancer cachexia to improve patient symptoms, functionality and quality of life.

### Preclinical evidence for anti‐growth differentiation factor 15 therapy in cancer cachexia

In a variety of human and mouse GDF‐15 secreting tumour models, tumour implantation caused an increase in circulating GDF‐15 levels, resulting in a decline in body weight (fat and skeletal muscle lean mass), food intake, skeletal muscle strength (force generation), physical activity (home cage locomotion and voluntary wheel running), exercise tolerance (treadmill running) and survival in animals, which were all reversed by GDF‐15/GFRAL neutralizing antibodies, including ponsegromab.^8^, ^17^, ^18^, ^19^ When co‐administered with the chemotherapy agent cisplatin, ponsegromab reversed both tumour and cisplatin‐driven body weight loss without interfering with the cisplatin antitumor effect in mice.^8^ Ponsegromab also mitigated cisplatin‐induced emesis and anorexia in monkeys.^8^

### Preliminary phase 1 insights on ponsegromab in cancer cachexia

Ponsegromab was first evaluated in two phase 1 single‐dose studies in 71 healthy adult participants (53 received ponsegromab and 18 received placebo).^20^, ^21^ Single SC doses of ponsegromab were well tolerated by study participants when administered SC at 0.1–300 mg in these studies—all treatment‐emergent adverse events (TEAEs) were mild, with only four treatment‐related adverse events (AEs) reported in three participants that were all related to mild injection site reactions. In these studies, serum unbound GDF‐15 concentrations were suppressed to below the lower limit of assay quantification within hours of a single SC administration of ponsegromab at doses ≥1 mg.

Ponsegromab was next evaluated in a phase 1b first‐in‐patient, open‐label, single‐arm study (NCT04299048) of ponsegromab of 200 mg SC every 3 weeks for a maximum of 12 weeks/5 doses in 10 participants with advanced metastatic NSCLC, advanced/unresectable PANC or metastatic CRC, cachexia and elevated circulating levels of GDF‐15 of ≥1.5 ng/mL.^22^ Median serum unbound GDF‐15 concentration was suppressed to below the lower limit of assay quantification (0.0424 ng/mL) from 3 h post‐dose on Day 1 through Week 15 (3 weeks after the final dose). Moreover, participants showed a 4.63‐kg (standard error: 1.98) gain in weight compared with baseline, over a 12‐week ponsegromab treatment period, with no AEs or serious AEs (SAEs) that were considered related to ponsegromab.^22^ There was also some preliminary evidence of improvements in exploratory endpoints of appetite and physical activity. Findings in this study must be considered in the context of the very small sample size and open‐label design.

A further phase 1b study of ponsegromab was started in patients with advanced cancer, anorexia and elevated circulating levels of GDF‐15 ≥1.5 ng/mL, randomized to either ponsegromab of 200 mg SC every 3 weeks or placebo during a 6‐week double‐blind period followed by an optional 18‐week open‐label treatment period^23^; however, this study was terminated early to focus on the current phase 2 study after enrolment of only 18 participants, less than half of the planned sample size. The median percentage reduction from baseline in unbound GDF‐15 concentrations was 98% following ponsegromab during the 6‐week double‐blind phase. Only one TEAE (moderate AE of myalgia) was considered treatment related.

**Figure 1:**
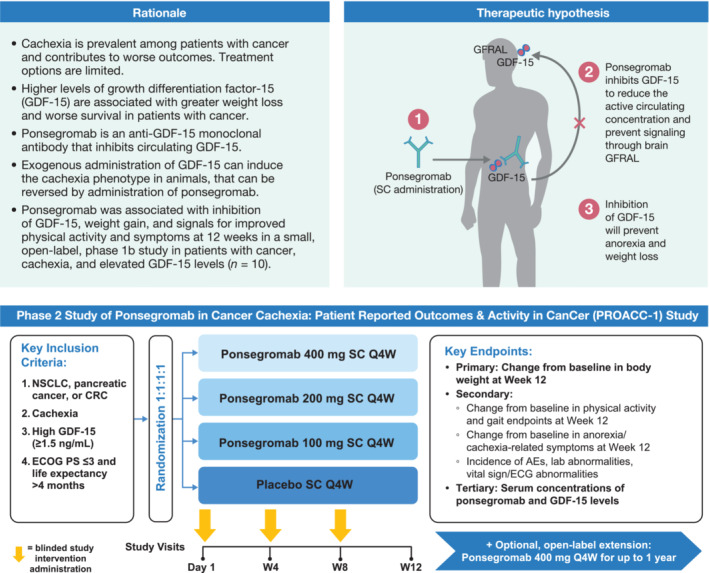
Central illustration. AE, adverse event; CRC, colorectal cancer; ECG, electrocardiogram; ECOG PS, Eastern Cooperative Oncology Group performance status; GFRAL, glial cell‐derived neurotrophic factor family receptor α‐like; NSCLC, non‐small‐cell lung cancer; Q4W, every 4 weeks; SC, subcutaneous; W, Week.

Overall, the combination of the observational data implicating GDF‐15 involvement in cancer cachexia, the preclinical evidence supporting the potential therapeutic relevance of targeting GDF‐15 in amelioration of cancer cachexia and the preliminary phase 1 insights summarized above provided sufficient rationale for this phase 2 PROACC‐1 study (*Figure* *1*).

## Methods

### Study design

The primary objective of the PROACC‐1 study is to assess the effect of ponsegromab compared with placebo on body weight in participants with cancer, cachexia and elevated concentrations of GDF‐15. Secondary objectives are to assess the effect of ponsegromab compared with placebo on physical activity, physical function, actigraphy, symptoms (including anorexia/appetite, nausea and vomiting, and fatigue) and safety. Exploratory objectives will assess the pharmacokinetics, pharmacodynamics, Response Evaluation Criteria in Solid Tumors (RECIST), change in lumbar skeletal muscle index (LSMI) and immunogenicity of ponsegromab.

**Figure 2:**
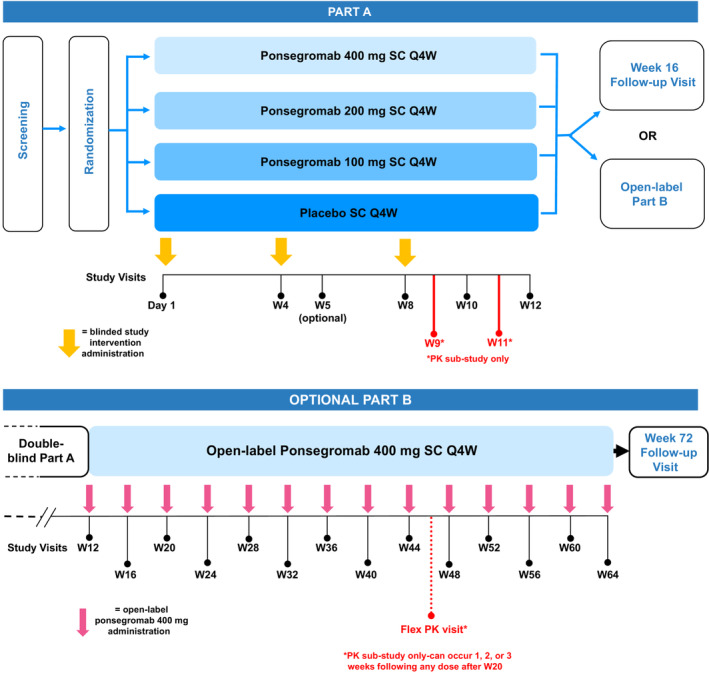
Study design. PK, pharmacokinetics; Q4W, every 4 weeks; SC, subcutaneous; W, Week.

The study will be conducted in two parts (*Figure* *2*). The study design includes input from patients with cancer who had significant unintentional weight loss and other symptoms of cachexia, and their caregivers. Part A is a double‐blind, placebo‐controlled, 12‐week treatment period in which participants will be randomly assigned (1:1:1:1) to ponsegromab (100, 200 or 400 mg) or matching placebo, administered by SC injection every 4 weeks, for a total of 3 doses. Given the potential for platinum‐based chemotherapy to induce GDF‐15,^8^ randomization will be stratified by presence/absence of background platinum therapy.

7Part B of the study is an optional, open‐label treatment extension with ponsegromab of 400 mg administered SC every 4 weeks for up to 1 year. Participants opting to continue to the open‐label period will receive their first dose of open‐label ponsegromab of 400 mg at the Week 12 visit (the last visit of the double‐blind period). Upon completion of the open‐label period, there will be a follow‐up visit at Week 72. Participants who do not proceed to the open‐label period will complete the Week 12 visit with a follow‐up visit at Week 16. A pharmacokinetic substudy will be conducted in a subset of participants in Part A, with additional samples (two during Part A and one during Part B) obtained for more comprehensive pharmacokinetic/pharmacodynamic evaluation in patients with cancer and cachexia.

The study will be conducted in accordance with the principles stated in the Declaration of Helsinki (1996), the International Conference on Harmonization Good Clinical Practice guideline and local and national regulations. The study protocol has been reviewed and approved by an institutional review board or independent ethics committee for each participating clinical research centre. All participants will provide signed informed consent.

### Participant selection

Key eligibility criteria are provided in *Table* *1*. Eligible participants must have an active diagnosis of NSCLC, CRC or PANC. Cachexia will be diagnosed according to the weight loss–BMI components of the International Consensus Criteria (body mass index [BMI] <20 kg/m^2^ with involuntary weight loss of >2% within 6 months prior to screening or involuntary weight loss of >5% within 6 months prior to screening irrespective of BMI)^2^ and elevated circulating levels of GDF‐15 (≥1.5 ng/mL) as measured using the Roche Elecsys® GDF‐15 assay (Roche Diagnostics, Germany)^24^ at screening. The sarcopenia–weight loss component of the International Consensus Criteria for cachexia (i.e., appendicular skeletal muscle index consistent with sarcopenia [males <7.26 kg/m^2^; females <5.45 kg/m^2^] and any degree of weight loss >2%)^2^ was not incorporated into inclusion criteria as appendicular skeletal muscle index is not available for most participants in clinical practice. Key exclusion criteria include current active reversible causes of decreased food intake (e.g., oral mucositis and mechanical obstructions), receiving tube feedings or parenteral nutrition at screening or randomization, cachexia caused by other reasons (e.g., chronic obstructive pulmonary disease [COPD] and heart failure), major surgery within 4 weeks prior to randomization or major surgery planned during the study.

**Table 1:** Key eligibility criteria

Key inclusion criteria
Participants aged ≥18 years
Documented histologic or cytologic active diagnosis of NSCLC, PANC or CRC receiving/completed standard‐of‐care treatment
Cachexia defined by BMI–weight loss components of the International Consensus Criteria^a^
Elevated GDF‐15 levels (≥1.5 ng/mL [as measured using the Investigational Use Only Roche Elecsys GDF‐15 assay] at screening)
For Part A of the study, ECOG PS ≤3 and life expectancy of ≥4 months

Key exclusion criteria
Current active reversible causes of decreased food intake (e.g., oral mucositis and mechanical obstructions)
Receiving tube feedings or parenteral nutrition at screening or randomization
Cachexia caused by other reasons (e.g., COPD, heart failure or AIDS)
Major surgery within 4 weeks prior to randomization or major surgery planned during the study
Initiation of new treatment with systemic glucocorticoids within 4 weeks prior to randomization
Concurrent administration with anamorelin hydrochloride, megestrol acetate, cannabinoids, olanzapine or mirtazapine, if prescribed for purpose of increasing appetite/body weight, within 30 days prior to randomization
Concurrent investigational products within 30 days or 5 half‐lives (whichever is longer) of the first dose of study intervention
Concurrent administration of a GLP‐1 receptor agonist‐based therapy, if prescribed specifically to promote weight loss, within 30 days prior to randomization
History of severe liver disease or cirrhosis, unrelated to metastatic cancer, or renal disease requiring dialysis

Abbreviations: BMI, body mass index; COPD, chronic obstructive pulmonary disease; CRC, colorectal cancer; ECOG PS, Eastern Cooperative Oncology Group performance status; GDF‐15, growth differentiation factor 15; GLP‐1, glucagon‐like peptide 1; NSCLC, non‐small‐cell lung cancer; PANC, pancreatic cancer.

^a^ BMI <20 kg/m^2^ with involuntary weight loss >2% within 6 months prior to screening or involuntary weight loss >5% within 6 months prior to screening irrespective of BMI.^2^

### Assessments and study endpoints

#### Change in weight

The primary study endpoint is change from baseline body weight at Week 12 in participants who received ponsegromab versus those who received placebo (Part A of the study). Change from baseline in body weight will also be assessed as an exploratory endpoint in Part B of the study, where data permit (*Table* *2*).

**Table 2:** Key endpoints

Study Part A
Primary
Change from baseline body weight at Week 12
Secondary
Change from baseline in FAACT subscale scores (FAACT‐ACS and FAACT‐5IASS) at Week 12
Change from baseline score for CRCSD questions related to anorexia/appetite, nausea and vomiting, and fatigue at Week 12
Change from baseline in physical activity and gait endpoints measured with remote digital sensors at Week 12
Incidence of adverse events and laboratory, vital sign and ECG abnormalities
Tertiary/exploratory
Serum concentrations of ponsegromab
Serum concentrations of GDF‐15
Incidence of antidrug antibodies and neutralizing antibodies
Tumour status according to RECIST 1.1 guidelines using CT scan at Week 12
Change from baseline in LSMI using CT scans at Week 12

Study Part B^a^
Tertiary/exploratory
Change from baseline body weight
Incidence of adverse events and laboratory, vital sign and ECG abnormalities
Serum concentrations of ponsegromab
Serum concentrations of GDF‐15
Incidence of antidrug antibodies and neutralizing antibodies

Abbreviations: 5IASS, anorexia‐related symptoms scale (five items within the ACS); ACS, anorexia and cachexia subscale; CRCSD, Cancer‐Related Cachexia Symptom Diary; CT, computed tomography; ECG, electrocardiogram; FAACT, Functional Assessment of Anorexia‐Cachexia Therapy; GDF‐15, growth differentiation factor 15; LSMI, lumbar skeletal muscle index; RECIST, Response Evaluation Criteria in Solid Tumors.

^a^ Study Part B endpoints will be evaluated as data permit.

#### Symptoms

Secondary endpoints will include patient‐reported outcome (PRO) measures, implemented using a sponsor‐provided electronic PRO device. Change from baseline in Functional Assessment of Anorexia‐Cachexia Therapy (FAACT) subscale scores will be assessed at Week 12 (secondary endpoint). FAACT is an instrument that combines the 27‐item Functional Assessment of Cancer Therapy‐General (FACT‐G) instrument and a 12‐item anorexia and cachexia subscale (ACS).^25^ Five items within the ACS comprise an anorexia‐related symptoms scale (5IASS). Together, the 39‐item FAACT measures physical, emotional, functional and social well‐being in the previous 7 days, as well as perceptions of appetite and weight. The instrument uses a 5‐point scale (0–4), whereby higher scores are associated with a higher health‐related quality of life.

Change from baseline score for Cancer‐Related Cachexia Symptom Diary (CRCSD) questions related to anorexia/appetite, nausea and vomiting, and fatigue will be assessed at Week 12. The CRCSD is a novel daily, self‐reported questionnaire that measures the severity or frequency of symptoms related to cancer cachexia (e.g., appetite, nausea and vomiting, and fatigue) over the previous 24 h using a numeric rating scale. This tool has been developed based on qualitative patient research along with a review of current literature and existing relevant measures. During development, 3 clinicians and 20 participants completed concept elicitation interviews and an additional 12 patients completed cognitive debriefing of newly developed items. Psychometric (quantitative) validation is planned once phase 2 data are available. Additional PRO assessments are also included as tertiary/exploratory endpoints.

#### Physical activity and function

Secondary endpoints will also include change from baseline to Week 12 in physical activity and gait endpoints measured using wearable, remote digital sensors (accelerometry) (*Table* *2*). One sensor will be placed on the lumbar region, and one sensor will be placed on the non‐dominant wrist. Monitoring will be conducted during screening, from Week 8 to Week 9 and from Week 10 to Week 12 for a minimum of 7 days. Participants will be asked to wear the sensor on the wrist continuously, and the sensor on the lumbar during the day only, during monitoring periods. Measurement parameters will include moderate to vigorous physical activity time, sedentary activity time, non‐sedentary activity time, total vector magnitude, mean activity level during maximum daily 6 min of activity, mean gait speed and 95th percentile gait speed (data permitting). Additional measurement parameters are included as tertiary/exploratory endpoints.

Physical function will also be separately evaluated using change from baseline at Week 12 in the following PROs: Patient‐Reported Outcomes Measurement Information System (PROMIS‐Physical Function), and Patient Global Impression of Severity (PGI‐S) and Change (PGI‐C)‐based assessments of physical function, physical activity and walking.

#### Pharmacokinetics, pharmacodynamics and immunogenicity

Exploratory endpoints during Part A and Part B (where data permit) of the study will include serum concentrations of ponsegromab and GDF‐15 (*Table* *2*). As ponsegromab is a monoclonal antibody, immunogenicity samples will be collected for the determination of antidrug antibodies and neutralizing antibodies. On ponsegromab dosing days in Part A and Part B, blood samples taken for pharmacokinetics, GDF‐15 and immunogenicity will be collected prior to administration of ponsegromab.

#### Safety and tolerability

TEAEs including monitoring for injection site reactions, SAEs, safety laboratory tests, vital signs and electrocardiogram (ECG) abnormalities are a secondary endpoint and will be monitored during Part A and Part B of the study (*Table* *2*). TEAEs and SAEs will be elicited/collected from informed consent through follow‐up.

#### Other assessments

Computerized tomography of chest, abdomen and pelvis will be acquired prior to randomization and at Week 12. A central imaging laboratory will determine skeletal muscle and adipose tissue areas at the level of the third lumbar vertebrae to derive percentage change from baseline in body composition parameters including LSMI at Week 12, which are included as exploratory endpoints. These computed tomography (CT) scans will also be used to explore the effect of ponsegromab compared with placebo on tumour status using the RECIST guidelines.^26^

### Randomization and statistical analyses

Approximately 168 participants will be enrolled: 42 randomly assigned each to placebo or ponsegromab of 100, 200 or 400 mg using a central randomization list. This number is expected to ensure that 120 evaluable participants complete Part A of the study. Analysis of the primary endpoint will be a Bayesian E_max_ model applied to Week 12 results from a mixed model repeated measures (MMRM) analysis including all post‐treatment timepoints up to Week 12. The Bayesian E_max_ model is anticipated to best describe the dose–response of ponsegromab.^27^ The MMRM model will include participant as a random term and baseline, time (as a factor), baseline‐by‐time interaction, treatment and treatment‐by‐time interaction as fixed terms in the model. Additional terms for type of therapy (i.e., platinum or not) and type of cancer will be fitted in the model. The Bayesian E_max_ model will include an informative placebo prior, based on historical results from relevant internal and external studies. Change from baseline in physical activity and gait endpoints, FAACT and CRCSD scores will be analysed separately using MMRM models and the censored analysis set. Least squares means (90% confidence interval [CI]) and mean differences versus placebo (90% CI and *P* value) will be provided. No adjustments will be made for multiplicity.

Safety data will be summarized descriptively. The safety analysis set is defined as all participants randomly assigned to study intervention and who take at least one dose of study intervention.

### Trial status

As of July 2023, the study is currently recruiting participants across sites in the United States, Canada, Japan, China, Taiwan, Australia, Spain, Slovakia, Poland and Bulgaria. Additional sites will be activated in Hungary.

## Discussion

Approximately half of patients with advanced cancer will develop cachexia.^1^ As a frequent and debilitating condition across cancer types, cachexia is an area of significant unmet medical need.^28^ The spectrum of cachexia is wide, ranging from precachexia (defined by anorexia, metabolic change and minor weight loss of ≤5%) to cachexia (weight loss >5% over the past 6 months, BMI <20 and any degree of weight loss ≥2%, or appendicular skeletal muscle index consistent with sarcopenia [males <7.26 kg/m^2^; females <5.45 kg/m^2^] and any degree of weight loss >2%) and refractory cachexia (active catabolism and lack of success with weight‐loss management strategies, low performance scores and <3 months of expected survival), with progressive depletion of energy stores and BMI combined with weight loss.^2^ Patients with cancer cachexia have increased risk of treatment‐related toxicity, reduced responsiveness to anticancer treatment and a reduced life expectancy.^1^, ^2^ Hence, there is an urgent need to develop pharmacologic interventions to slow or reverse the progression of cachexia as this may ultimately impact survival. The aim of this study is to support the clinical development of ponsegromab as a novel inhibitor of GDF‐15‐mediated signalling and potential therapy for cancer cachexia. Preliminary phase 1b data suggest that suppression of GDF‐15 with ponsegromab may lead to improvement in cachexia‐related symptom burden, building on promising preclinical observations in animal tumour models where neutralization of GDF‐15 reverses anorexia and weight loss and improves muscle function and survival.^8^, ^18^, ^19^, ^22^ Furthermore, given the observations of GDF‐15 elevation with platinum‐based chemotherapy,^8^ patients who receive standard‐of‐care antitumor treatment that includes systemic platinum‐based therapy may represent a specific population that could potentially garner greater benefit from GDF‐15 inhibition. Given the lack of established drug therapies for cancer cachexia, the design, implementation and analysis of the PROACC‐1 study will evaluate the potential role of ponsegromab in patients with cancer, cachexia and elevated concentrations of GDF‐15 and will help inform whether larger scale pivotal trials testing ponsegromab are warranted.

## Conflict of interest statement

JC: consulting or advisory role (Actimed, Aveo, BIO Alta, Enzychem, Faraday, G1 Therapeutics, Merck, Partner Therapeutics, Pfizer, Sandoz and Seagen); research funding (AstraZeneca, Helsinn Healthcare and Pfizer [to institution]); and stock or other ownership (Pfizer). ARS, DMB, IJ, JDG, JR, MAH, MIR, RQ, SLL and SMC: employment (Pfizer); and stock or other ownership (Pfizer).
